# Acute severe hepatitis and COVID-19: case report

**DOI:** 10.11604/pamj.2024.49.2.44247

**Published:** 2024-09-02

**Authors:** Saber Hmimass, Maryeme Kadiri, Mohamed Borahma, Fatima-Zahra Chabib, Camelia Berhili, Nawa Lagdali, Imane Ben Elbarhdadi, Fatima-Zahra Ajana

**Affiliations:** 1 Department of Hepato-Gastroenterology C, Ibn Sina Hospital, Mohamed V University, Rabat, Morocco

**Keywords:** COVID-19, severe acute hepatitis, case report

## Abstract

COVID-19, caused by SARS-CoV-2, has been declared an international public health emergency. Patients with COVID-19, even without a history of liver disease, frequently present with liver test disturbances. Due to the multisystemic involvement of COVID-19, the pathogenesis of liver injury is likely to be multifactorial, involving systemic inflammation, small vessel thrombosis, hepatic hypoxia, and potential drug toxicity, ruling out direct infection of hepatocytes by SARS-CoV-2. COVID-19 can cause severe acute hepatitis. We report the case of a 25-year-old man admitted to emergency with abdominal pain who presented with acute severe liver failure before respiratory signs.

## Introduction

Individuals diagnosed as COVID-19-positive may present with liver balance disturbances; however, cases of acute liver failure revealing COVID-19 are rare in the literature. We report the first case of a man with polymerase chain reaction (PCR) confirmed COVID-19 who presented with severe acute hepatitis before the onset of respiratory signs.

## Patient and observation

**Patient information:** a 25-year-old man came to the emergency department of the Ibn Sina Hospital in Rabat with abdominal pain. He didn´t have a past medical history of alcohol, hepatotoxic drugs, or medicinal plants, and he didn´t have close contact with someone who tested positive for COVID-19. Respiratory symptoms as a cough, sore throat, dyspnea or diarrhea were not present. On arrival, he presented with hepatic colic with early, spontaneous, postprandial vomiting, followed by bilious vomiting, and significant physical asthenia.

**At the clinical examination:** the patient was admitted to the hepato-gastroenterology department. He was conscious, well oriented in time and space; GCS 15/15, hemodynamically stable, apyretic, with no icterus or signs of hepatocellular failure. Abdominal examination showed a supple abdomen, without collateral venous circulation, homogeneous hepatomegaly of 18cm, no splenomegaly, and grade II ascites. The rest of the clinical examination was unremarkable, particularly the cardiovascular and pleuropulmonary examinations.

**Diagnostic procedure:** abdominal ultrasound showed an enlarged liver (FH at 20cm) with regular contours and a homogeneous echostructure with no focal lesions. The gallbladder was alithiasic with a thin wall, the intrahepatic and extrahepatic bile ducts were not dilated, the spleen was of normal homogeneous size, there was no venous circulation or shunt, and there was a moderate amount of peritoneal effusion. Biological results showed ASAT 1845 IU/L (normal value (NV): 34UI/L), ALAT 1323 IU/L (NV: 55 UI/L), PAL 224 IU/L (1.75*N), GGT 125 IU/L (2*N), total bilirubin 24mg/L and TP 33%. Serologies for hepatitis A, B, C, E, cytomegalovirus and Epstein-Barr virus, HCV PCR, and autoimmune markers were normal. On day 2, the temperature remained normal but respiratory signs appeared, including cough, sore throat, respiratory distress and the appearance of a mucocutaneous subicterus with normal-coloured stools and urine and atypical diarrhea (3-4 stools per day). Biological values worsened: AST 3961 UI/L, ALT 1802 UI/L, prothrombin rate at 31% and factor V 30%. A chest X-ray showed bilateral interstitial syndrome. Nasopharyngeal samples were taken and PCR for SARS-CoV-2 was positive.

**Therapeutic intervention:** the patient developed a psychomotor and verbal slowdown and was transferred to intensive care, where he was treated with hydroxychloroquine 200 mg*2/d combined with azithromycin 500mg and vitamin therapy (vitamin C + ZINC + vitamin D). Decrease in transaminases; AST 618 IU/L, ALT 491 IU/L, total bilirubin 30 mg/L but prothrombin rate at 26% (Table 1).

**Table 1:** evolution of liver biochemical profile of our patient

	At admission	H48 after	After treatment
Aspartate aminotransferase (UI/l)	1845	3961	618
Alanine aminotransferase (UI/l)	1323	1802	491
Total bilirubin (mg/l)	24	-	30
Prothrombin rate	33%	31%	26%
Factor V	-	30%	-

**Follow-up and outcomes:** unfortunately, the patient died of multivisceral, neurological, renal, and hepatic failure (total bilirubin 35 mg/L IHC: prothrombin rate at 19% and FV 29%) and gastrointestinal hemorrhage (haematemesis).

**Patient consent:** written informed consent has been obtained from the patient for the publication of this case report.

## Discussion

Coronavirus disease 2019, also known by the acronym COVID-19, is a zoonosis newly transmitted to humans, resulting from infection with a coronavirus named SARS-CoV-2. The first known occurrence of this infection was reported in a patient in Wuhan, China, on 17^th^ November 2019. The World Health Organization (WHO), following an initial international alert issued on 9^th^ January 2020, designated the COVID-19 outbreak a public health emergency of international concern [1]. Transmitted mainly by direct contact via respiratory droplets expelled when speaking, coughing, and sneezing, and less frequently via objects contaminated by these droplets, COVID-19 causes symptoms characteristic of acute respiratory illness. These symptoms include cough, fever, dyspnoea, and fatigue, often associated with a loss of smell or taste. The available data highlight the seriousness of this disease, especially when compared with seasonal influenza epidemics. The main cause of death associated with COVID-19 is acute respiratory distress, affecting between 30% and 50% of patients admitted to intensive care units [1]. The negative impact of COVID-19 on the field of hepatology has been clear. Patients with liver disease do not appear to be more common among those infected with COVID-19, suggesting that the presence of liver disease is not in itself a risk factor for SARS-CoV-2 infection. However, the morbidity and mortality associated with COVID-19 are increased in the presence of various concomitant pathologies, and certain liver diseases are no exception [2].

The pathogenic link is currently hypothetical, but it is conceivable that the chronic inflammation associated with metabolic dysfunction-associated fatty liver disease (MAFLD) facilitates the triggering of the cytokine storm characteristic of COVID-19 [3]. Steatosis associated with MAFLD appears to be associated with a severe course of pneumonitis. Several studies have highlighted how MAFLD, particularly in the presence of significant liver fibrosis, can influence the course of COVID-19, even in the complete absence of metabolic comorbidity [3]. Due to the multisystemic involvement of COVID-19, it is highly likely that the pathogenesis of liver injury is multifactorial. Factors such as systemic inflammation, small vessel thrombosis, hepatic hypoxia and possible drug toxicity could explain the changes observed, without the need to consider direct infection of hepatocytes by SARS-CoV-2, which remains unlikely [1]. It has been reported that up to 60% of SARS patients have liver test abnormalities [4]. A recent epidemiological study showed that 43 cases of COVID-19 had varying degrees of liver function abnormalities and increased levels of alanine aminotransferase (ALT) or aspartate aminotransferase (AST), and 1 in 99 patients with COVID-19 had severe liver damage [5]. Liver function abnormalities in patients with COVID-19 were mainly manifested by abnormal ALT or AST levels, with a slight increase in bilirubin levels. In a study of 69 patients by Wang *et al*. [6], 23 had elevated ALT (33%) and 19 had elevated AST (28%). In the Chinese study [7], 44 of the 298 patients (14.8%) had liver damage, and those with severe liver damage (36.2%) were more prone to these elevations than patients with mild liver damage (9.6%). According to the study by Cai *et al*. [8], the incidence of liver injury could be as high as 78% among 82 deaths due to laboratory-confirmed SARS-CoV-2 infection.

Based on a review of the literature and the opinions of experts in India, hepatitis cases suddenly decreased when COVID-19 infection rates fell, but increased when the number of cases was high, so COVID-19 infection may increase the incidence of hepatitis in some people, but these hepatitis cases are not as serious. However, other researchers believe that COVID-19 could have caused dozens of cases of unexplained severe hepatitis between April and July 2021 [4]. Current studies have shown that poor prognosis in patients with COVID-19 is related to gender (male), age (60 years), and underlying disease (hypertension, diabetes, cardiovascular disease). It was found that there was no independent correlation between ALT, AST, total bilirubin, alkaline phosphatase, albumin, and other indicators of liver function, and severe COVID-19, indicating that the liver was not the main target organ [9]. However, ALT, AST, total bilirubin, and other liver function indices were significantly increased in severe COVID-19 patients compared with mild COVID-19 patients, and liver function indices gradually returned to normal during recovery. Liver damage in patients with mild COVID-19 is often temporary and can return to normal without any special treatment. Our patient had mild pulmonary involvement with severe hepatic involvement with transaminases greater than 50 times normal with hepatocellular prothrombin rate at 28% and factor V at 30%.

## Conclusion

Liver damage is common in patients with COVID-19, and may range from a slight disturbance of the liver balance to severe hepatitis. Close attention should be paid to the state of liver function in patients with COVID-19.
